# Impact of diabetes mellitus on adenolymphangitis episodes in patients with filarial lymphedema—Towards an integrated care approach

**DOI:** 10.1371/journal.pntd.0013486

**Published:** 2025-09-08

**Authors:** Nikita Kubal, Sowmiya Manavalan, Vijayakumar Balakrishnan, Nileshkumar Elangovan, Danapriyaa Dharmalingam, Vijesh Sreedhar Kuttiatt

**Affiliations:** 1 Unit of Clinical and Molecular Medicine, ICMR-Vector Control Research Centre (VCRC), Indira Nagar, Puducherry, India; 2 Department of Global Health, Georgetown University, Washington, DC, United States of America; Universidad de la República Uruguay: Universidad de la Republica Uruguay, URUGUAY

## Abstract

**Background:**

Filarial lymphedema, caused by lymphatic filariasis, is characterized by chronic swelling and recurrent skin infections. Acute adenolymphangitis (ADL) episodes significantly exacerbate morbidity. Diabetes mellitus (DM) increases susceptibility to infections; however, the relationship between diabetes and ADL frequency and severity in filarial lymphedema patients remains unclear. This study investigated the influence of diabetes on ADL attacks and identified predictors influencing ADL episodes in patients with filarial lymphedema.

**Methodology/Principal findings:**

This case-control study analyzed data from 166 patients (79 diabetic, 87 non-diabetic) attending a filariasis management clinic in Puducherry, India. Sociodemographic data, clinical characteristics, ADL frequency, severity, and adherence to morbidity management and disability prevention (MMDP) practices were collected. Univariate and multivariate logistic regression analyses examined the association between diabetes mellitus and ADL episodes. Diabetes status did not significantly influence ADL frequency or severity. However, lymphedema severity and intertrigo presence emerged as significant predictors of increased ADL attack risk. Home-based care was associated with increased ADL frequency, suggesting potential inadequacies in current self-care practices.

Integration of filarial lymphedema care into existing non-communicable disease (NCD) clinics at primary healthcare centers (PHCs) and subcenters could streamline care delivery, enhance patient management, and optimize healthcare resource utilization.

**Conclusions:**

This study emphasizes the importance of integrated care approaches addressing both diabetes and lymphedema management. Lymphedema severity and intertrigo management should be prioritized in patient care to mitigate ADL risks. Strengthening home-care education and protocols is essential for improving ADL outcomes. Future research should explore structured community-based and prospective studies to clarify diabetes management’s potential protective role and further enhance the quality of care for affected individuals.

## Author summary

Lymphatic filariasis causes filarial lymphedema, a chronic condition characterized by swelling and recurrent skin infections known as acute adenolymphangitis (ADL). Diabetes mellitus (DM) is associated with increased susceptibility to infections, raising concerns about its potential impact on ADL episodes. This study examined whether diabetes influences ADL frequency or severity among patients with filarial lymphedema in Puducherry, India. Contrary to expectations, diabetes mellitus did not significantly affect ADL outcomes, although advanced lymphedema and intertrigo significantly increased ADL risk. Additionally, home-based care was linked to more frequent ADL episodes, suggesting the need for improved home-care practices. Foot care is the common point in diabetes and lymphedema care. These findings highlight the necessity for integrated healthcare approaches and better education on self-care management to reduce ADL attacks and improve patient outcomes.

## Introduction

Filarial lymphedema, commonly referred to as elephantiasis, is a chronic manifestation of lymphatic filariasis (LF) [1]. LF is a neglected tropical disease (NTD) prevalent in over 72 countries worldwide, with the highest burden in India, Indonesia, Nigeria, and Bangladesh [2]. The condition results from chronic dysfunction of the lymphatic system due to infection from filarial parasites *Wuchereria bancrofti, Brugia malayi, or Brugia timori* [3]. Once established, lymphedema is irreversible and often worsens over time, leading to physical disfigurement, recurrent skin infections, and significant psychosocial consequences [4]. Delays and diagnostic challenges, often stemming from limited healthcare infrastructure and overlapping clinical presentations with other chronic conditions such as diabetes mellitus (DM), complicate timely diagnosis and management.

Filarial lymphedema pathogenesis involves the dilation of lymphatic vessels due to mechanical obstruction by adult worms, as well as an inflammatory immune response that leads to fibrosis and chronic lymphatic dysfunction [5]. The damage to lymphatic vessels disrupts fluid drainage, leading to progressive swelling of the affected limbs. Patients frequently experience recurrent episodes of acute adenolymphangitis (ADL), characterized by sudden fever, chills, severe pain, and localized limb inflammation, which significantly impacts quality of life and contributes to disease progression [6]. Despite the importance of ADL in disease exacerbation, the immunological mechanisms underlying susceptibility remain poorly understood, especially regarding interactions with comorbid conditions [7].

For individuals already affected by filarial lymphedema, morbidity management and disability prevention (MMDP) are essential for reducing disease progression and ADL frequency. The WHO’s guidelines for lymphedema include rigorous limb hygiene, prevention of secondary infections, and patient education on self-care practices [8]. Key aspects include regular washing with soap and water, careful drying and moisturizing to prevent intertrigo and bacterial entry points. Other components, such as exercise, limb elevation, and proper footwear, aim to prevent fluid accumulation and reduce the risk of recurrent infections [9]. However, despite MMDP’s effectiveness, many patients continue to suffer from frequent ADL episodes, suggesting that additional host-related factors contribute to susceptibility [10].

Diabetes mellitus (DM) is an emerging global health challenge, significantly increasing infection susceptibility through hyperglycemia-induced impairment of immune responses, including reduced leukocyte function, compromised cytokine production, and weakened skin barrier integrity [11,12]. India, facing one of the highest global diabetes burdens, has over 77 million adults living with diabetes as of 2019 [13]. The prevalence is projected to increase significantly in the coming decades [14,13]. Rapid urbanization, sedentary lifestyles, unhealthy diets, and genetic predisposition contribute to the rising incidence of diabetes mellitus in India [15,13]. In a recent study by ICMR, it was found that Puducherry has the second-highest rate of prevalence of diabetes in India, with a rate of 26.3 percent of cases [16]

Diabetes mellitus profoundly affects health complications such as cardiovascular disease, peripheral arterial disease, chronic kidney disease, retinopathy, and neuropathy, all exacerbating infection susceptibility [11]. Diabetic foot is a major concern for diabetic patients, significantly increasing morbidity and mortality [17]. It is characterized by poor blood circulation, nerve damage, and impaired wound healing[18]. This significantly elevates the risk of skin and soft tissue infections (SSTIs) [17]. Insights from diabetic complications provide essential context for understanding infection risk in patients with filarial lymphedema.

Both diabetes and filarial lymphedema independently compromise circulation and immune function through distinct pathological processes. At a molecular level, chronic hyperglycemia in diabetes and lymphatic dysfunction in filarial lymphedema may interact synergistically to exacerbate immune dysfunction and skin barrier integrity, potentially increasing ADL frequency and severity. Therefore, effective management requires a multidisciplinary approach, addressing both metabolic control and lymphedema care to prevent infections and improve patient outcomes. Enhanced clinical awareness and targeted interventions can mitigate the combined impact of these diseases, reducing the burden on patients and healthcare systems.

Lymphatic filariasis and diabetes mellitus are significant public health challenges, particularly in endemic regions like India. MDA (Mass Drug Administration) has effectively reduced LF prevalence through preventative chemotherapy; however, it does not alleviate symptoms or improve outcomes in individuals who have already contracted LF and developed chronic lymphedema [19]. Diabetes further complicates the clinical picture, increasing the risk of severe infections and exacerbating lymphedema. Comprehensive care models integrating diabetes management with lymphedema care are essential for improving patient outcomes and reducing the burden of these interconnected conditions.

A significant gap remains in research examining the association between diabetes and acute adenolymphangitis (ADL) episodes in patients with filarial lymphedema. This study aims to investigate the frequency and severity of ADL episodes among diabetic and non-diabetic filarial lymphedema patients. Clarifying this relationship could support the development of targeted therapeutic interventions and improved integrated care protocols, ultimately enhancing patient outcomes, and inform public health strategies in regions with a high prevalence of both conditions.

## Methods

### Ethics statement

The study was approved by the Institute Human Ethics Committee (IHEC-0222/N/F), and written informed consent was obtained from the participants before data collection.

### Study design and setting

This study utilized a case-control design to examine the relationship between diabetes and the occurrence and severity of acute adenolymphangitis (ADL) attacks in individuals suffering from filarial lymphedema. The research was conducted at the Filariasis Management Clinic, located within the Vector Control Research Centre (VCRC) in Puducherry, India. The study population consisted of patients diagnosed with filarial lymphedema for more than two years and residing within a 25 km radius of the VCRC clinic. This geographic criterion was established to maintain a uniform study population and account for potential healthcare access differences that could impact ADL frequency. The clinic serves a diverse patient demographic, including individuals from varying socioeconomic backgrounds, different age groups, and semi-urban settings. The inclusion of patients from these backgrounds enhances the generalizability of findings while allowing for subgroup analyses based on economic and demographic factors.

Patients with filarial lymphedema were divided into two groups: the case group, consisting of individuals with diabetes, and the control group, comprising non-diabetic individuals.

Participants were recruited from the clinic’s patient records, ensuring that all selected individuals had a confirmed diagnosis of lymphedema and had received care at the clinic. Sampling was performed using convenience sampling, with patients sequentially selected based on clinic record availability and meeting the inclusion criteria. Diabetes status was confirmed through both self-reported history and verification via medical records.

To account for potential differences in care settings, home visits were conducted in addition to clinic-based assessments. These home visits were not conducted for all participants but rather for a representative subset of clinic attendees. Each patient selected for a home visit received one structured visit by trained field workers, and data collected during home visits were cross-referenced with clinic-based reports to ensure accuracy. Health supplies, including soap and antiseptics, were provided to patients, and vital signs were recorded to assess overall health status.

### Sample size

The desired sample size for each group (diabetic and non-diabetic) was calculated using the Fleiss with Continuity Correction method on OpenEpi. Parameters for the calculation included a two-sided significance level (1-alpha) of 0.05, power (1-beta) of 80%, a ratio of controls to cases of 1:1, a proportion of controls with exposure (P0) of 0.10 (indicating 10% of non-diabetic patients are expected to develop recurrent skin and soft tissue infections (SSTIs)), and a proportion of cases with exposure (P1) of 0.30 (indicating 30% of diabetic patients are expected to develop recurrent SSTIs). Although the required sample size was 114, the study enrolled 166 participants (79 diabetic, 87 non-diabetic) to enhance statistical power and account for potential ethical considerations, possible dropouts, or incomplete data collection.

### Data collection

Data was collected through detailed questionnaires administered at the clinic and during home visits. Before the main data collection, the questionnaire was pilot tested at the clinic to assess clarity and cultural appropriateness. Based on feedback, the questionnaire was refined by adding detailed questions on ADL attacks and removing redundant queries related to diabetes management. The questionnaires covered demographic details, personal history, lymphedema-related information, co-morbidities, and ADL attack frequency and severity. The case group questionnaire included additional sections specific to diabetes, capturing detailed information on diabetes management and complications. Translators assisted in the process to ensure accuracy, and home visits also involved taking vital signs and providing health supplies to the patients.

Filarial lymphedema severity was classified according to Dreyer’s seven-stage classification system, which aligns with the WHO’s grading system. Patients were categorized into three grades: Grade 1/2, representing early-stage lymphedema with mild swelling and reversible changes; Grade 3, indicating moderate lymphedema with mild swelling and reversible changes; Grade 4, signifying severe, irreversible lymphedema with thickened skin and possible elephantiasis. Grades 1 and 2 were analyzed together due to their clinical similarity in early-stage lymphedema progression, supported by existing literature [20,21].

### Statistical analysis

A total of 156 participants were included in the final analysis. Data were coded and analyzed using STATA 18.0 BE. Descriptive statistics were used to summarize participant characteristics, with categorical variables analyzed using Chi-square (χ²) tests. Continuous variables were assessed using t-tests for normally distributed data and Wilcoxon rank-sum tests for skewed distributions. Statistical significance was set at p < 0.05 for all comparisons. ADL attack frequency was evaluated across two time periods: within the last year and over the entire two-year period. Patients were categorized by lymphedema grade (1/2, 3, or 4), and ADL frequency was compared between diabetic and non-diabetic groups using Chi-square and Wilcoxon rank-sum tests.

ADL severity was assessed using predefined clinical indicators, including hospitalization, antibiotic injections received, impact on daily routine (e.g., work), and episode duration of seven or more days. A binary severity variable was created to classify patients based on the number of severe ADL attacks. Univariate and multivariate logistic regression analyses were conducted to assess predictors of ADL attacks. Independent variables included diabetes status, lymphedema grade, socioeconomic status (SES), MMDP adherence, age, gender, and the presence of intertrigo. The dependent variable was the presence or absence of ADL attacks, and odds ratios (OR) with 95% confidence intervals (CIs) were calculated. Model fit was assessed using the Hosmer-Lemeshow test, and discriminative ability was evaluated using the area under the ROC curve (AUC).

To further examine care disparities, the Wilcoxon rank-sum test was used to compare ADL attack intervals between home-based care and clinic-based care settings, identifying whether home-based care resulted in significantly different ADL frequency or severity. This analysis was conducted to clarify if observed differences in ADL episodes were due to variations in care accessibility or adherence.

## Results

This study examined the relationship between diabetes status and the occurrence and severity of acute adenolymphangitis (ADL) episodes in 166 patients with filarial lymphedema, including 79 diabetic and 87 non-diabetic participants. Statistical analyses were conducted to compare ADL frequency, severity, lymphedema grade, and adherence to morbidity management and disability prevention (MMDP) practices between the two groups.

### Sociodemographic characteristics

Sociodemographic characteristics stratified by diabetes status are summarized in Table 1. Age distribution was similar between diabetic and non-diabetic groups, with 65.8% and 67.6% aged 60 years or older, respectively (p = 0.821). Gender distribution did not differ significantly, although there were more females among non-diabetics (67.5%) compared to diabetics (55.3%; p = 0.116).

**Table 1:** Sociodemographic characteristics of the study population stratified by diabetes status.

Variable	Diabetes		P-value*
	Yes (n = 76)	No (n = 80)	
Age (in years)			
< 60	26(34.2%)	26(32.4%)	0.821
≥ 60	50(65.8%)	54(67.6%)	
Gender			
Female	42(55.3%)	54(67.5%)	0.116
Male	34(44.7%)	26(32.5%)	
Education			
High school and above	27(35.5%)	21(26.3%)	0.210
Below High school	49(64.5%)	59(73.8%)	
Occupation			
Employed	29(38.2%)	23(28.8%)	0.213
Unemployed	47(61.8%)	57(71.3%)	
SES			
Upper	–	–	0.853
Upper middle	4(5.3%)	6(7.5%)	
Lower middle	23(30.3%)	23(28.8%)	
Upper lower	41(54.0%)	40(50.0%)	
Lower	8(10.5%)	11(13.8%)	
SES			
Lower	49(64.5%)	51(63.8%)	0.925
Middle	27(35.5%)	29(36.3%)	

*Note: *Pearson’s chi-square test was applied for categorical variable comparisons.*

Educational attainment did not show significant differences; 64.5% of diabetic participants and 73.8% of non-diabetics had below high school education (p = 0.210). Employment status was also comparable, with unemployment rates at 61.8% among diabetics and 71.3% among non-diabetics (p = 0.213).

Socioeconomic status (SES), evaluated both categorically (five-level) and dichotomously, was similar between the two groups. Using a simplified SES classification, lower SES was reported by 64.5% of diabetic participants and 63.8% of non-diabetics (p = 0.925).

### Co-morbidities and LF characteristics

Table 2 summarizes the distribution of comorbid conditions according to diabetes status. Hypertension was significantly more prevalent among diabetic participants (56.6%) compared to non-diabetic participants (36.3%; p = 0.011).

**Table 2:** Co-morbidity and LF characteristics of the participants*.

Variable	Diabetes		P-value**
	Yes (n = 74)	No (n = 83)	
Intertrigo presence	36(47.4%)	38(47.5%)	0.987
Presence of other co-morbidity*	45(59.2%)	39(48.8%)	0.190
Hypertension	43(56.6%)	29(36.3%)	**0.011**

*Note: *The presence of other co-morbidities refers to patients indicating on their questionnaire that they suffered from other diseases separate from diabetes such as cardiac illness, hyperlipidemia, hypertension, thyroid disorders, etc.*

**Note: *Pearson’s chi-square test was applied for categorical variable comparisons.*

There were no significant differences between diabetic and non-diabetic groups in terms of intertrigo presence (47.4% vs. 47.5%; p = 0.987) or other reported comorbid conditions, such as cardiac disease, hyperlipidemia, thyroid disorders, and additional non-diabetic illnesses (59.2% vs. 48.8%; p = 0.190).

### ADL attack frequency and severity

Table 3 summarizes the differences in ADL attack patterns and lymphedema-related characteristics between diabetic and non-diabetic participants. Although a higher proportion of non-diabetic participants reported experiencing ADL attacks (56.3%) compared to diabetic participants (46.1%), this difference was not statistically significant (p = 0.203).

**Table 3:** Lymphedema grades and ADL attacks, showing the frequency and duration of ADL attacks, stratified by diabetes status.

Variable	Diabetes		P-value*
	Yes (n = 74)	No (n = 82)	
Lymphedema grade			
Grade 1/2	42(55.3%)	44(55.0%)	0.502
Grade 3	17(22.4%)	23(28.8%)	
Grade 4	17(22.4%)	13(16.3%)	
LF duration			
≤ 10	14(18.4%)	20(25.0%)	0.366
10 – 20	18(23.7%)	25(31.3%)	
20 – 30	19(25.0%)	16(20.0%)	
> 30	25(32.9%)	19(23.8%)	
Experienced ADL attacks	35(46.1%)	45(56.3%)	0.203
Experienced ADL attacks in the last year	32(91.4%)	43(95.6%)	0.449
≥ 2 attacks	19(59.4%)	30(69.8%)	0.350
Experienced 4 or more ADL attacks over 2 years	10(28.6%)	20(44.4%)	**0.146**
Average ADL interval (in days)			
Mean (Q1 – Q3)	145.9 (73.6 – 183)	117.3 (76 – 152)	0.281
No severe episodes	2 (5.7%)	8(17.8%)	
Patients with 1 or more severe episodes	33(94.3%)	37(82.2%)	0.106
Number of severe ADL episodes	96	104	0.427
Mean(SD)	2.74(2.81)	2.31(2.03)	
% of episodes that were severe out of total ADL attacks Mean(SD)	83.3% (30.8)	74.0% (39.0)	0.247
Patients with severe ADL episodes			
≤1	17(48.6%)	23(51.1%)	0.822
≥ 2	18(51.4%)	22(48.9%)	
Average ADL episode duration (Days)			
< 7 days	22(62.9%)	24(53.3%)	0.393
≥ 7 days	13(37.1%)	21(46.7%)	

*Note: *Pearson’s chi-square test was applied for categorical variable comparisons, while continuous variables were compared using Student’s t-test.*

The frequency of having four or more ADL episodes over two years was also higher among non-diabetics (44.4%) than diabetics (28.6%), but this difference was not statistically significant (p = 0.146). Diabetic participants reported a longer average interval between ADL attacks (mean: 145.9 days) compared to non-diabetics (mean: 117.3 days), though this difference did not reach statistical significance (p = 0.281).

Regarding severe episodes, there was no significant difference in the proportion of severe episodes out of total ADL attacks between diabetic (83.3%) and non-diabetic participants (74.0%; p = 0.247). Similarly, there was no significant difference in the mean number of severe ADL episodes (2.74 ± 2.81 for diabetics vs. 2.31 ± 2.03 for non-diabetics; p = 0.427).

Duration of ADL episodes was comparable between groups, with no significant difference between diabetic and non-diabetic participants experiencing episodes lasting ≥7 days (37.1% vs. 46.7%; p = 0.393). Lastly, no significant differences were observed in lymphedema grade distribution (p = 0.502) or LF duration categories (p = 0.366).

### MMDP adherence

Adherence to morbidity management and disability prevention (MMDP) practices was comparable between diabetic and non-diabetic participants, with no statistically significant differences observed for any of the self-care practices assessed (p > 0.05 for all comparisons).

The majority of participants in both groups reported practicing limb washing (79.0% of diabetics vs. 82.5% of non-diabetics; p = 0.544) and limb elevation (89.5% vs. 92.5%; p = 0.509). Similarly, nail and interdigital area care was reported by 61.8% of diabetic participants and 67.6% of non-diabetics (p = 0.148). Use of limb bandaging (32.9% vs. 37.5%; p = 0.547) and limb massage (27.6% vs. 33.8%; p = 0.408) was also similar between groups.

Overall, these findings suggest no significant difference in adherence to MMDP practices between individuals with and without diabetes Table 4.

**Table 4:** MMDP Practices- comparisons between diabetic and non-diabetic participants.

MMDP Practices	Diabetes		P-value*
	Yes (n = 74)	No (n = 82)	
Do you wash your limbs?			
Yes	60(79.0%)	66(82.5%)	0.544
Do you wash your nails and interdigital areas?			
Yes	47(61.8%)	54(67.55%)	0.148
Do you elevate your limbs?			
Yes	68(89.5%)	74(92.5%)	0.509
Do you bandage your limbs?			
Yes	25(32.9%)	30(37.5%)	0.547
Do you massage your limbs?			
Yes	21(27.6%)	27(33.8%)	0.408

*Note: *Pearson’s chi-square test was applied for categorical variable comparisons.*

### Predictors of ADL attacks

Univariate logistic regression analysis identified several factors associated with the likelihood of experiencing ADL attacks (Table 5). Intertrigo emerged as a strong and significant predictor, with participants who reported intertrigo having nearly six times higher odds of ADL attacks compared to those without (OR: 5.14, 95% CI: 2.59–10.19, *p* < 0.001).

**Table 5:** Univariate Logistic Regression Analysis Associated with ADL Attacks.

Variable	OR (95% CI)	P-value*
Age (in years)		
< 60	1.00	–
>=60	0.76(0.39 – 1.49)	0.428
Gender		
Male	1.00	–
Female	0.98(0.51 – 1.86)	0.935
Education		
High school and above	1.00	–
Below High school	1.55(0.78 – 3.07)	0.201
Occupation		
Employed	1.00	–
Unemployed	1.08(0.55 – 2.10)	0.821
SES		
Lower	1.00	–
Middle	0.66(0.34 – 1.27)	0.214
Intertrigo		
No	1.00	–
Yes	5.14(2.59 – 10.19)	**<0.001**
co-morbidity		
No	1.00	–
Yes	1.10(0.59 – 2.07)	0.767
Hypertension		
No	1.00	–
Yes	1.12(0.60 – 2.10)	0.730
Diabetes		
No	1.00	–
Yes	0.66(0.35 – 1.25)	0.202
Lymphedema grade (Worst-case)		
Grade 1/2	1.00	–
Grade 3	3.13(1.43 – 6.86)	**0.004**
Grade 4	4.64(1.85 – 11.64)	**0.001**
LF duration (Worst-case)		
<=10	1.00	–
10 – 20	0.73(0.30 – 1.82)	0.503
20 – 30	0.83(0.32 – 2.16)	0.704
> 30	0.53(0.22 – 1.32)	0.173

* **Note:** *Univariate logistic regression analysis was performed. Odds ratios (OR) and 95% confidence intervals (CI) are reported. Statistical significance was set at p < 0.05.*

Lymphedema severity was also significantly associated with increased odds of ADL attacks. Participants with grade 3 lymphedema had over three times the odds compared to those with grade 1/2 (OR: 3.13, 95% CI: 1.43–6.86, p = 0.004), and those with grade 4 had nearly five times higher odds (OR: 4.64, 95% CI: 1.85–11.64, p = 0.001).

Diabetes showed a non-significant trend toward reduced odds of ADL attacks (OR: 0.66, 95% CI: 0.35–1.25, p = 0.202). Socioeconomic status (SES) was not significantly associated (OR: 0.66, 95% CI: 0.34–1.27, p = 0.214). Age, gender, education, occupation, presence of other comorbidities, hypertension, and LF duration were not significantly associated with ADL attack occurrence (p > 0.05 for all).

### Multivariate logistic regression

Multivariable logistic regression analysis was conducted to identify independent predictors of ADL attacks. As shown in Table 6, the presence of intertrigo remained a strong and significant predictor, with individuals affected by intertrigo having over three times greater odds of experiencing ADL attacks compared to those without (OR: 3.69, 95% CI: 1.65–8.23, p = 0.001).

**Table 6:** Multivariate Logistic Regression Analysis of Factors Associated with ADL Attacks.

Variable	OR (95% CI)	P-value*
Intertrigo		
No	1.00	–
Yes	3.69 (1.65 – 8.23)	**0.001**
Diabetes		
No	1.00	–
Yes	0.60(0.30 – 1.21)	0.155
Lymphedema grade (Worst-case)		
Grade 1/2	1.00	–
Grade 3	1.70(0.69 – 4.14)	0.246
Grade 4	2.26(0.78 – 6.56)	0.133

*Note: *Odds ratios (OR) and 95% confidence intervals (CI) derived from multivariate logistic regression analysis, adjusting for all variables presented. Statistical significance is defined at p < 0.05.*

Although diabetes showed a trend towards a lower likelihood of ADL attacks, this association was not statistically significant. Diabetic participants had 40% lower odds of reporting ADL episodes compared to non-diabetics (OR: 0.60, 95% CI: 0.30–1.21, p = 0.155).

Lymphedema severity exhibited an increasing trend of association with ADL attacks, although this was not statistically significant. Participants with grade 3 lymphedema had higher odds of ADL attacks (OR: 1.70, 95% CI: 0.69–4.14, p = 0.246), and those with grade 4 had more than twice the odds (OR: 2.26, 95% CI: 0.78–6.56, p = 0.133), compared to participants with grade 1/2.

### Model performance

The logistic regression model had an AUC of 0.728, indicating good discriminative ability, with a sensitivity of 66.25%, a specificity of 71.05%, and an overall classification accuracy of 68.6%. The Hosmer-Lemeshow test showed no evidence of poor fit (p = 0.765).

### Additional findings

The Wilcoxon rank-sum test indicated that patients receiving home-based care experienced more frequent ADL attacks than those treated in clinical settings (p = 0.037). Thus, underscoring the importance of timely clinical intervention in reducing ADL attack frequency.

These results highlight the complex interplay between diabetes status, lymphedema severity, and adherence to management practices in influencing ADL occurrence and severity.

## Discussion

This study investigated the relationship between diabetes status and the frequency and severity of acute adenolymphangitis (ADL) episodes in patients with filarial lymphedema. By analyzing demographic, socioeconomic, and clinical variables, we aimed to understand how these factors interact with diabetes status to influence ADL outcomes. Contrary to our initial hypothesis, diabetes was not significantly associated with ADL attacks. Although diabetic patients tended to report fewer ADL episodes and longer intervals between episodes, these differences did not reach statistical significance in univariate or multivariate analyses. This finding may be attributed to increased contact with healthcare systems among diabetic patients, including routine wound care, structured education, and consistent monitoring, which may help mitigate the risk of ADL episodes, though this hypothesis warrants further investigation due to non-significant findings in the current study.

However, despite comparable numbers of ADL episodes, diabetic patients experienced ADL episode durations similar to those of non-diabetic patients, contrary to initial expectations based on the impaired immune function commonly observed in individuals with diabetes [10,9]. These findings suggest that factors influencing ADL duration may be independent of diabetes status. The results highlight the importance of integrated care approaches that address both diabetes management and lymphedema care to minimize the risk and impact of ADL.

Lymphedema grade emerged as a strong predictor of ADL attack frequency and severity. Patients in more advanced stages (grades 3 and 4) were at significantly higher risk, supporting the need for early detection and intervention to prevent disease progression and reduce the burden of ADL. Targeted screening and proactive lymphedema management may be critical in high-risk populations. This aligns with existing literature on lymphedema severity in ADL outcomes [9].

Intertrigo emerged as a significant predictor of ADL attacks, reinforcing its role as a key modifiable risk factor. Patients with intertrigo were over four times more likely to report ADL episodes, underscoring the role of secondary bacterial infections as triggers for ADL. Routine skin and timely treatment of intertrigo should be emphasized in MMDP programs to reduce ADL risk. This highlights the need for routine skin assessments in lymphedema care programs and targeted interventions to manage intertrigo before it leads to complications. Treating intertrigo early could reduce the risk of ADL in filarial lymphedema patients, warranting further investigation into how these factors interact [22].

Furthermore, adherence to MMDP practices did not significantly differ between diabetic and non-diabetic groups. The proportions of patients adhering to essential practices such as limb washing, elevation, bandaging, and massage were comparable across both groups (p > 0.05 for all comparisons). This suggests that diabetes status does not markedly influence adherence behavior, possibly due to uniform care protocols and patient education. However, MMDP practices were assessed only once during the study and based on self-reported oral responses, rather than through multiple follow-up visits, which may not accurately reflect adherence over time. Future studies should incorporate multiple assessments to better evaluate adherence trends and long-term compliance [23].

Finally, patients who managed ADL episodes at home experienced shorter intervals between attacks, suggesting that home-based care may be less effective than clinical care in managing LF-related complications. This may be due to inadequate infection control, lack of access to antibiotics, or delays in seeking medical attention. These findings emphasize the need for improved patient education on recognizing early signs of ADL, strengthening home-care protocols, and ensuring timely access to clinical interventions. Strengthening community-based healthcare services could provide much-needed support to patients who are unable to access clinic-based treatment regularly.

### Study limitations and future directions

The retrospective study design introduces recall bias, as participants reported ADL episodes over two years. A prospective study design would allow for real-time data collection and more accurate measurement of ADL frequency and severity. Additionally, the clinic-based sampling approach may not fully represent the broader community population, as patients who regularly attend clinic services may have better health care access and adherence to treatment, potentially influencing the findings. Future research should incorporate community-based sampling to ensure more generalizable results.

Although logistic regression analysis was used to adjust for potential confounders, residual confounding factors may persist, such as differences in healthcare-seeking behavior, medication adherence, environmental conditions, and dietary factors that could still influence the results. Future studies should include more comprehensive assessments of socioeconomic status and healthcare access to determine how these factors interact with ADL risk.

Finally, given that home-based care was associated with increased ADL attack frequency, future research should evaluate the effectiveness of different home-care strategies and identify gaps in patient education or resource availability. Studies comparing outcomes between structured clinic-based care and home-based management models could provide valuable insights into optimizing ADL prevention efforts.

## Conclusion

This study provides novel insights into the interplay between diabetes and ADL attacks in filarial lymphedema patients. Contrary to our initial hypothesis, diabetes was not significantly associated with the occurrence of ADL attacks. Although diabetic patients appeared to experience fewer ADL episodes and longer intervals between episodes, these differences did not reach statistical significance. Further investigation is needed to explore whether structured healthcare access, routine monitoring, and wound care practices among diabetic patients may confer any protective effects against ADL episodes.

Additionally, episode durations were comparable between diabetic and non-diabetic individuals, suggesting that any hypothesized influence of diabetes on delayed wound healing or immunological compromise did not manifest as significantly prolonged ADL episodes in this study. These findings point to a nuanced interaction between disease processes that requires integrated, multidisciplinary care strategies.

The analysis reinforced the significance of lymphedema severity and intertrigo as key predictors of ADL frequency and severity. Patients with more advanced lymphedema (grades 3 and 4) and those with intertrigo were at significantly increased risk, emphasizing the need for early detection, proactive lymphedema care, and timely skin infection management.

The association between home-based care and increased ADL attack frequency suggests that current home management practices may be insufficient. Improvements in patient education, clearer home-care protocols, and better access to clinical resources are critical to optimizing ADL outcomes.

Looking ahead, future research should prioritize community-based sampling to enhance generalizability and adopt prospective designs to reduce recall bias and improve temporal accuracy. Interventional studies are also needed to assess the impact of early intertrigo management and explore the effectiveness of clinic-based versus home-based treatment models.

Finally, addressing barriers to healthcare access, particularly among diabetic patients managing filarial lymphedema at home, will be essential in shaping effective, equitable public health interventions. Health systems should invest in integrated diabetes management and early LF intervention. There is significant scope for integrating diabetes and lymphedema care into existing NCD clinics at primary health centers (PHC) and subcenters. Training Auxiliary Nurse Midwives (ANMs) in this integrated care approach is crucial, which represents an important programmatic implication of this study in India. A strengthened collaboration with the National Program for Prevention and Control of Cancer, Diabetes, Cardiovascular Diseases, and Stroke (NPCDCS) can facilitate this integration and enhance service delivery. These strategies can reduce the combined burden of diabetes and filarial lymphedema, ultimately improving these vulnerable populations’ quality of life and clinical outcomes.

## Supporting information

S1 Data De-identified dataset. (XLSX)
